# New insecticide screening platforms indicate that Mitochondrial Complex I inhibitors are susceptible to cross-resistance by mosquito P450s that metabolise pyrethroids

**DOI:** 10.1038/s41598-020-73267-x

**Published:** 2020-10-01

**Authors:** Rosemary S. Lees, Hanafy M. Ismail, Rhiannon A. E. Logan, David Malone, Rachel Davies, Amalia Anthousi, Adriana Adolfi, Gareth J. Lycett, Mark J. I. Paine

**Affiliations:** 1 Vector Biology Department, Liverpool School of Tropical Medicine, Liverpool, L3 5QA UK; 2 Innovative Vector Control Consortium, Liverpool School of Tropical Medicine, Liverpool, L3 5QA UK

**Keywords:** Enzymes, Entomology

## Abstract

Fenazaquin, pyridaben, tolfenpyrad and fenpyroximate are Complex I inhibitors offering a new mode of action for insecticidal malaria vector control*.* However, extended exposure to pyrethroid based products such as long-lasting insecticidal nets (LLINs) has created mosquito populations that are largely pyrethroid-resistant, often with elevated levels of P450s that can metabolise and neutralise diverse substrates. To assess cross-resistance liabilities of the Complex I inhibitors, we profiled their susceptibility to metabolism by P450s associated with pyrethroid resistance in *Anopheles gambiae* (CYPs 6M2, 6P3, 6P4, 6P5, 9J5, 9K1, 6Z2) and *An. funestus* (CYP6P9a). All compounds were highly susceptible. Transgenic *An. gambiae* overexpressing CYP6M2 or CYP6P3 showed reduced mortality when exposed to fenpyroximate and tolfenpyrad. Mortality from fenpyroximate was also reduced in pyrethroid-resistant strains of *An. gambiae* (VK7 2014 and Tiassalé 13) and *An. funestus* (FUMOZ-R). P450 inhibitor piperonyl butoxide (PBO) significantly enhanced the efficacy of fenpyroximate and tolfenpyrad, fully restoring mortality in fenpyroximate-exposed FUMOZ-R. Overall, results suggest that in vivo and in vitro assays are a useful guide in the development of new vector control products, and that the Complex I inhibitors tested are susceptible to metabolic cross-resistance and may lack efficacy in controlling pyrethroid resistant mosquitoes.

## Introduction

Recent years have seen a plateauing of malaria cases, following halving of malaria fatalities in Africa between 2000–2016. The disease is transmitted to humans by *Anopheles* mosquitoes and much of this success can be attributed to the use of long-lasting insecticide-treated nets (LLINs)^1^. The mainstay of net insecticides has been members of the pyrethroid class of voltage-gated sodium channel (Vgsc) modulators, due to their rapid knockdown effect against mosquito vectors and low toxicity to humans^2^. The recent small rises in malaria have partly been attributed to pyrethroid resistance evolving rapidly in the major malaria vectors^3^ with sixty-six malaria-endemic countries reporting confirmed resistance to this class of insecticide since 2010^4^. The predominant resistance mechanisms to pyrethroid insecticides in malaria vectors are selection of target site insensitivity of the Vgsc, known as knockdown resistance (kdr), and metabolic resistance resulting from increased insecticide detoxification that is most often mediated by cytochromes P450^5,6^.

**Figure 1:**
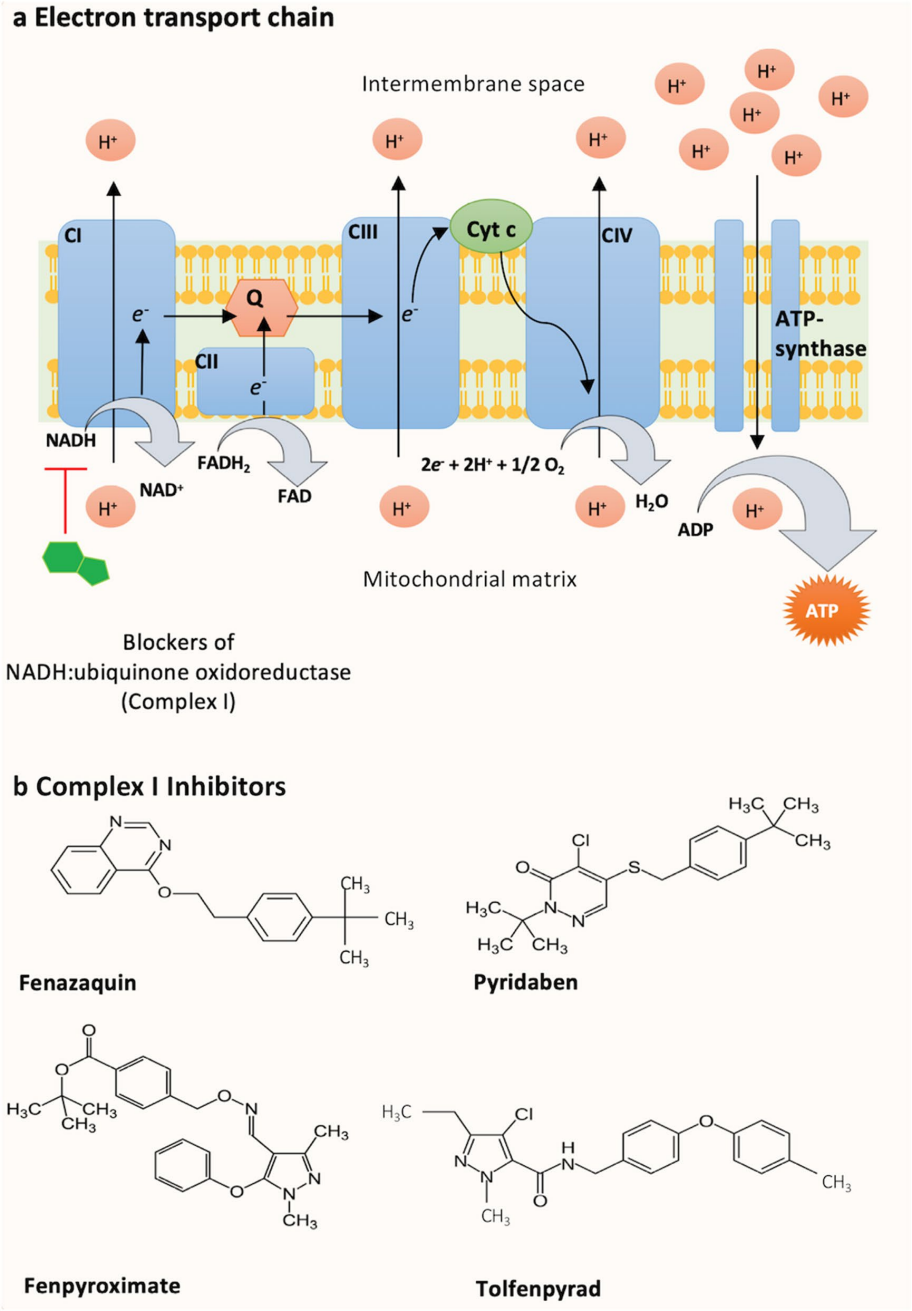
Schematic diagram of the mitochondrial electron transport chain (**a**) and structures of the Complex I inhibitors used in this study (**b**). NADH donates two electrons to Complex I (CI), which are transferred to complexes 2–4 (CII–CIV) via quinone (Q) and cytochrome c (Cyt c) co-factors and redox reactions to generate a proton gradient (H^+^) that drives ATP synthesis. Complex I inhibitors act by disrupting ATP synthesis as a result of the inhibition of NADH:ubiquinone oxidoreductase activity.

Introducing insecticides with alternative modes of action to pyrethroids is critical to mitigate the current resistance issues in mosquito populations^2^. One of the ways to accelerate compounds through to utilisation in public health is through repurposing existing agricultural pesticides for use in appropriate formulations in mosquito control^7–9^. Indeed, a recent screen of 30,000 leads from agriculture chemistries against *An. stephensi* identified 12 promising chemistries for the control of adult mosquitoes, including complex I inhibitors^10^. Mitochondrial Complex I inhibitors are a structurally diverse group of synthetic insecticides and acaricides that disrupt arthropod respiration by interfering with proton-translocating NADH:ubiquinone oxidoreductase (EC 1.6.5.3; Complex I) activity. This results in the blockade of mitochondrial oxidative phosphorylation and reduced production of ATP (Fig. 1a)^11,12^. Active ingredients from quinazoline (Fenazquin), pyridazinone (Pyridaben) and pyrazole (Fenpyroximate and Tolfenpyrad) groups (Insecticide Resistance Action Committee (IRAC) Mode of Action Class 21A) (Fig. 1b) are used against agricultural pests and could potentially be used for malaria control. Tolfenpyrad, for instance, has been found effective against *Anopheles gambiae, An. arabiensis* and *Culex quinquefasciatus* when used with attractive toxic sugar baits^13^. Recently, all four compounds were included in a screen of pesticides for potential for use against malaria vectors conducted for the Innovative Vector Control Consortium^8^ (IVCC) and with the exception of fenazaquin all were shown to be active against *An. gambiae* exposed via topical application and tarsal contact in the presence of an adjuvant. Tolfenpyrad and fenpyroximate were further shortlisted for consideration as ingredients in new vector control products on the basis of favourable bioefficacy against pyrethroid susceptible *An. gambiae*^8^.

Given the high levels of pyrethroid resistance in Africa, an important step in the roll-out of alternative chemistries for use against Anopheline populations is to examine their efficacy against pyrethroid resistant mosquitoes and to assess the potential for cross-resistance to pre-existing detoxifying mechanisms in the target populations. Recently, an experimental hut trial was conducted in an area of pyrethroid-resistant *An. gambiae* s.l., and *An. funestus* s.s. to identify the efficacy of a non-pyrethroid insecticide-treated durable wall lining (ITWL)^14^. Despite high mortality of pyrethroid resistant strains in lab contact bioassays, a polypropylene material containing abamectin (a macrocyclic lactone targeting chlorine channels) and fenpyroximate demonstrated low efficacy of the ITWL in hut trials which may be attributed to failure of mosquitoes to land on treated surfaces^14^. However potential cross-resistance issues to one of these compounds could not be excluded since they were used as a mixture.

Mitochondrial Complex I inhibitors are most commonly used as acaricides and there have been numerous reports of resistance including pyridaben, fenpyroximate and tebufenpyrad in the two-spotted spider mite, *Tetranychous urticae*^15^, as well as cross-resistance between Complex I inhibitors^16,17^, which has been associated with elevated levels of cytochrome P450 (P450 or CYP) activity^17,18^. P450s catalyse the mono-oxygenation of a wide range of xenobiotics, which can lead to detoxification of insecticides in arthropods^6^. Many African populations of adult Anopheline mosquitoes express elevated levels of P450 activity associated with metabolic co-resistance to pyrethroids^19^ and other insecticide classes. Since P450s can metabolise a wide range of chemical substrates these pose a pre-eminent cross-resistance liability for new chemistries introduced into the insect control market^20,21^. *An. gambiae* CYP6P3 and CYP6M2 and *An. funestus* CYP6P9a and CYP6P9b are amongst the P450s most commonly found to be overexpressed in pyrethroid-resistant populations and have been demonstrated to metabolise a range of insecticide classes in vitro including pyrethroids, juvenile hormones, organophosphates and carbamates^20,21^.

Previous work has produced a bank of recombinant P450s commonly overexpressed in pyrethroid-resistant populations of *An. gambiae* (CYPs 6M2, 6P2, 6P3, 6P4, 6P5, 9J5, 9K1, 6Z2) and *An. funestus* (CYP6P9a)^21^ to screen for metabolic activity in vitro. In addition, the Gal4 UAS system has been used to overexpress CYP6P3 and CYP6M2^22,23^ in *An. gambiae* for phenotypic in vivo assessment of P450 metabolism by these key pyrethroid resistance marker genes. Here, we have combined the use of in vitro and transgenic screening, with bioassays on established pyrethroid-resistant strains of *An. gambiae* and *An. funestus* with known metabolic resistance markers to assess potential cross-resistance liabilities of Complex I inhibitors. This demonstrated a useful suite of tests that can be applied to other compounds to guide the development of new insecticide-based vector control products.

## Results

### In vitro metabolic cross-resistance profile of complex I inhibitors

In order to examine the susceptibility of Complex I inhibitors to metabolism by common P450 markers of pyrethroid resistance, fenazaquin, pyridaben, fenpyroximate and tolfenpyrad (Fig. 1b) were screened against eight *An. gambiae* P450s (CYPs 6M2, 6P2, 6P3, 6P4, 6P5, 9J5, 9K1, 6Z2) and one *An. funestus* P450 (CYP6P9a). A substrate depletion assay commonly used for the measurement of drug metabolism was used that is less sensitive than metabolite formation but facilitates rapid screening of multiple compounds. Thus we applied the 20% substrate depletion cut-off value that is normally applied in drug screening to clearly distinguish substrate depletion from weak or uncertain metabolism and baseline variability^24^.

**Figure 2:**
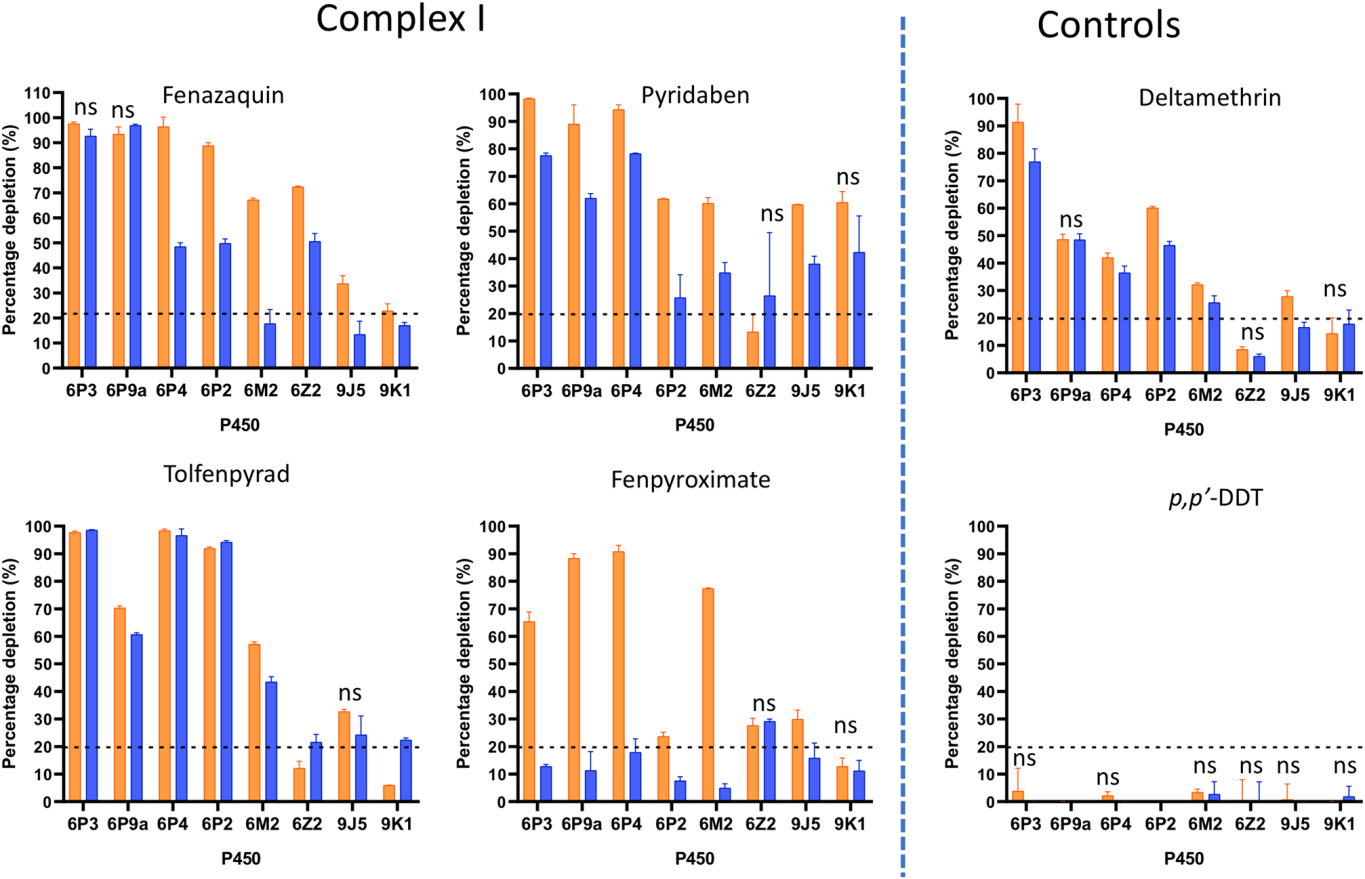
Complex I inhibitor metabolism by *Anopheles* P450s. Bars represent the proportion (% depletion) of 10 μM insecticide cleared by 0.05 µM P450 with (0.4 µM) or without b5 in the presence of NADPH. Values and significance levels are given in Supplementary Table 1. Error bars represent standard deviation (N = 3). NS, no significant difference in insecticide clearance +/− b5. Complex I and control compounds are indicated. Orange bars, +b5; blue bars, − b5..

Results of this assay are presented in Fig. 2 and Supplementary Table 1. All four Complex I inhibitors were highly susceptible to metabolism by the P450 panel tested (Fig. 2 and Supplementary Table 1). Three compounds, fenazaquin, pyridaben and tolfenpyrad, were metabolised by all P450s in the presence and absence of b5. However, fenpyroximate metabolism was highly dependent on the presence of b5 (Fig. 2 and Supplementary Table 1) with all P450s except for CYP6Z2 (28 and 29% depletion in the presence and absence of b5, respectively).

As expected from previous work^21^, no turnover was observed with DDT for any P450, while deltamethrin was metabolised above threshold levels by all P450s except for CYPs 6Z2 and 9K1. Since CYP9K1 is known to have a slow rate of deltamethrin metabolism^25^ , and deltamethrin depletion (18 and 14% + /− b5 respectively) was below the cut-off threshold (20%), we expect this to reflect very slow rather than lack of metabolism. By contrast CYP6Z2 was not expected to metabolise deltamethrin as previous association with pyrethroid resistance has been primarily linked with the secondary metabolism of deltamethrin metabolites resulting from primary P450 and carboxyl-esterase cleavage (3-phenoxybenzoic alcohol and 3-phenoxybenzaldehyde)^26,27^. However, it was included as it is often overexpressed in pyrethroid-resistant populations.

### Analysis of transgenic lines overexpressing single P450 genes

**Figure 3:**
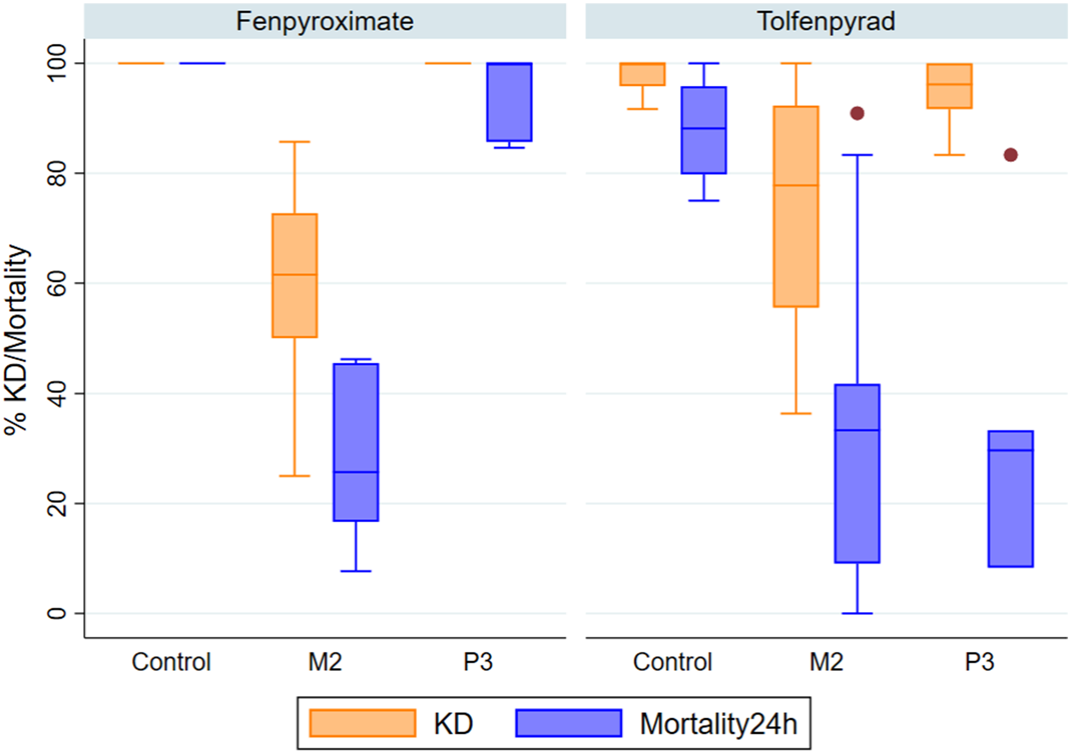
Knock down and mortality of two transgenic strains of *Anopheles gambiae*, overexpressing CYP6M2 (M2) and CYP6P3 (P3), exposed to fenpyroximate or tolfenpyrad in a glass tarsal plate assay, compared to a heterozygous A10 Gal strain (Control). Knock down at the end of exposure to a dried deposit of 125 mg/m^2^ of insecticide and 100 mg/m^2^ of the adjuvant RME applied to a glass petri dish in acetone, and mortality at 24-h post-exposure, are shown for the three strains. Box plots represent the median (centre line), 25th and 75th percentiles (box) and upper and lower adjacent values (whiskers) of 6 replicates , each replicate containing 10 mosquitoes, and dots represent outlier values.

Both fenpyroximate and tolfenpyrad have been shortlisted for further consideration in new vector control products^8^, and thus became the focus for in vivo studies. These were assayed against recently generated transgenic Gal4/UAS *An. gambiae* lines that overexpress the main P450s, CYP6P3 or CYP6M2 in multiple tissues, and demonstrate resistance to the pyrethroids, permethrin and deltamethrin in WHO bioassays^23^. These lines were assayed for sensitivity to fenpyroximate and tolfenpyrad in tarsal assays at fivefold concentration of LD95 in control mosquitoes obtained previously in Lees et al. 2019^8^. Mosquitoes overexpressing CYP6P3 displayed significantly reduced (p < 0.05) mortality after exposure to tolfenpyrad compared to controls (32 ± 21% vs 87 ± 10%), whereas no significant difference (p > 0.05) in sensitivity to fenpyroximate was observed (95 ± 6% vs 100%) (Fig. 3). In contrast, overexpression of CYP6M2 in mosquitoes significantly decreased (p < 0.05) mortality to both fenpyroximate (27 ± 12% vs 100%) and tolfenpyrad (35% ± 17 vs 87% ± 10) (Fig. 3).

It should be noted that all the mosquitoes displayed higher % knockdown after 60 min than mortality 24 h after exposure to tolfenpyrad and fenpyroximate, implying recovery from initial effects of the insecticide. However, limited difference in mortality was observed between 24 and 48 h post exposure to either compound (Supplementary Table 2).

### Analysis of colonised resistant ***An. gambiae*** strains

Analysis of sensitivity to tolfenpyrad and fenpyroximate was further assayed in colonised pyrethroid resistant mosquitoes known to overexpress specific P450s compared to susceptible strains^28^.

**Figure 4:**
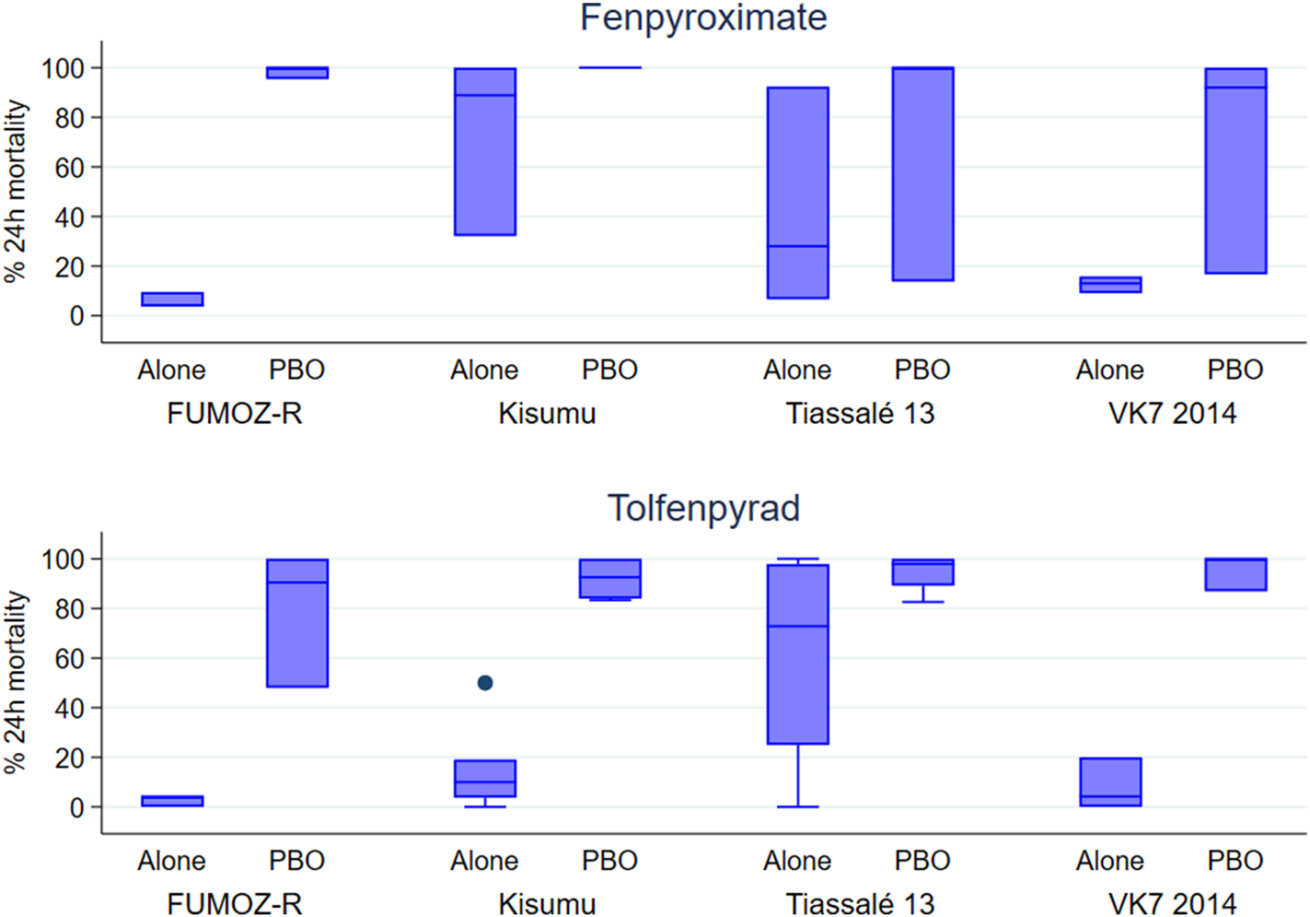
Mortality in 4 strains of *Anopheles* females 24 h after exposure in a CDC bottle bioassay to fenpyroximate or tolfenpyrad, alone or with the addition of PBO. Female adults were exposed the insecticides, with or without PBO, applied in acetone to the inside of a glass bottle, around 25 adults per bottle and 3 replicate bottle per treatment and strain. Bottles were coated with 160.8 µg per bottle of fenpyroximate or 143.84 µg per bottle of tolfenpyrad, with or without 400 µg of PBO. Box plots represent the median (centre line), 25th and 75th percentiles (box) and upper and lower adjacent values (whiskers) of 3 replicates, and dots represent outlier values.

In order to screen for cross-resistance *in colonised* pyrethroid resistant *mosquito* strains, Tiassalé 13, VK7 2014 and FUMOZ-R were chosen because they were known to overexpress specific P450s compared to susceptible strains^28^. *Cyp6m2, Cyp6p3* and *Cyp6p4* are highly upregulated in Tiassalé 13 and VK7 2014 strains of *An. gambiae*, and *Cyp6p9a* and *Cyp6p9b* are highly upregulated in the FUMOZ-R strain of *An. funestus* compared to the susceptible strains^28^. These strains were exposed to fenpyroximate and tolfenpyrad^8^ in a CDC bottle bioassay^29^ and mortality at 24 h post-exposure was compared to that of the susceptible Kisumu strain (Fig. 4). Each insecticide was tested either in the absence or presence of the P450 inhibitor, piperonyl butoxide (PBO), a synergist which is used to estimate the contribution of P450 metabolism to resistance phenotypes in pyrethroid resistant strains^30^.

In the absence of PBO, FUMOZ-R and VK7 2014 mortality 24 h post-exposure to fenpyroximate were significantly lower than Kisumu (6 ± 4% and 13 ± 4%, respectively vs 73 ± 41%) while the reduction in mortality in Tiassalé 13 (42 ± 50%) was not significant (Fig. 4). The results obtained with tolfenpyrad were highly inconsistent. The mortality in Kisumu exposed to tolfenpyrad was not the complete lethality expected with exposure to the LC_95_ established with this strain in a preliminary experiment (15 ± 15%); the mortality observed was also highly variable between replicates, although significantly higher in Tiassalé 13 (61 ± 46%) and not significantly different between FUMOZ-R (3 ± 3%) or VK7 2014 (8 ± 12%) and Kisumu.

In the parallel bioassays performed with bottles coated with the same concentrations of insecticide but with the addition of PBO, despite substantial variation between some replicates, PBO significantly (p < 0.001) enhanced 24-h mortality in all treatments and all strains (Fig. 4).

Fenpyroximate is a fast-acting insecticide^8^. In many of the treatments in the PBO assay there was some recovery of mosquitoes which were knocked down at the end of exposure but alive 24 h later (Supplementary Table 3). Tolfenpyrad was slower acting in comparison, with less than 10% difference in any treatments between knock down and mortality, except for some recovery by 24 h in Kisumu without the addition of PBO. Again, as observed with the transgenic lines, the increase in mortality from 24 to 48 h post-exposure was limited (less than 10% in any treatment, Supplementary Table 3).

Median mortality in the negative controls, consisting of unexposed and PBO only exposed mosquitoes, was less than 10% over all experiments, and there was no significant increase in mortality (p > 0.05) with the addition of PBO in controls (Supplementary Fig. 1). Pyrethroid resistance was confirmed in the three compounds by their significantly lower mortality on exposure to permethrin control bottles than Kisumu (p < 0.05). The addition of PBO to the permethrin controls significantly increased mortality (p < 0.05) in all strains except Kisumu, where mortality in both cases was 100% (Supplementary Table 2 and Supplementary Fig. 1), generally consistent with the reported involvement of metabolic mechanisms in the pyrethroid resistance of these strains^28^.

## Discussion

The results presented indicate that many P450s that metabolise pyrethroids also metabolise Complex I inhibitors in the malaria vectors *Anopheles gambiae* and *An. funestus*. The highest levels of metabolism were found in the CYP6P family members and CYP6M2, which are most frequently overexpressed across mosquito species endemic in Africa, reinforcing the suggestion that there is broad substrate compatibility of these enzymes and their potential for causing cross-resistance^20,21^. More selective metabolism was observed with CYP6Z2, CYP9J5 and CYP9K1; fenazaquin showed susceptibility to CYP6Z2 attack, while pyridaben was susceptible to CYP9J5 and CYP9K1 metabolism. Although the in vitro data is indicative of potential metabolic cross-resistance liabilities, in vivo detoxification that might lead to resistance is dependent on complex physiological factors. These include tissue localisation of P450s and interactions with NADPH-cytochrome P450 reductase (CPR) and b5, which modulate the rate of P450 activity in vitro^31^ and in vivo^32,33^. Fenpyroximate turnover by nearly all P450s assayed was highly dependent on the presence of b5, which may indicate that this electron transporter partner plays a significant in vivo role in the fenpyroximate resistance observed in the transgenic and wild type mosquitoes. This is further supported by the observation that b5 is commonly found to be upregulated in resistant strains^32^.

The transgenic lines offer the possibility to delineate the role individual P450 enzymes play in cross resistance phenotypes, since their genetic background is of susceptible mosquitoes and they overexpress a single P450. Complex I resistance phenotypes were conferred by overexpression of either of the two main insecticide metabolising P450s in *An. gambiae*. These mosquitoes express the transgene P450s in a broad tissue pattern and at overall levels that are greater than wild type strains^23^. Although not necessarily at physiologically relevant levels, they provide a good indication that these P450s could confer cross-resistance to Complex I inhibitors in pyrethroid resistant wild strains that also express these enzymes at elevated levels. From the transgenic data, it would appear that cyp6M2 has activity against both fenpyroximate and tolfenpyrad, whereas cyp6P3 has reduced activity against the latter compound. In recombinant protein assays both enzymes metabolise fenpyroximate, but only when b5 cofactor is added to the reaction. It may be possible then that the CYP6P3/CPR complex doesn’t associate readily with b5 in vivo, however more research is required to investigate this.

The assays in colonised pyrethroid resistant mosquitoes gives a direct indication of the potential for cross resistance to impact mosquito control, but cannot delineate which mechanism may be involved. The Tiassalé 13 and VK7 2014 strains of *An. gambiae* assayed constitutively overexpress CYP6P3, CYP6P4 and CYP6M2, as well as carry high frequencies of kdr resistance^28^. The VK7 2014 line is also thought to have a cuticular resistance mechanism that also contributes to insecticide resistance. The Anopheles lines also overexpress SAP2, which has recently been shown to confer pyrethroid resistance via a potential sequestration mechanism^34^. In contrast, *An. funestus* FUMOZ-R overexpresses CYP6P9a and CYP6P9b at up to 70 fold levels above susceptible strains^35,36^ and do not possess kdr alleles, and thus provide a direct assay of P450 involvement in cross-resistance. As predicted from in vitro and transgenic assays, all strains showed some degree of cross-resistance to fenpyroximate. FUMOZ-R was most highly resistant to fenpyroximate with mortality fully restored (> 98%) by a P450 inhibitor, PBO, as expected from a P450 dominant resistance mechanism. PBO also increased fenpyroximate potency in Tiassalé 13 and VK7 2014, consistent with elevated levels of CYP6P3, CYP6P4 and CYP6M2. However, PBO synergism was not complete, suggesting the potential contribution of other mechanisms described above towards cross-resistance to Complex I inhibitors in these *An. gambiae* strains. These results echo studies with the agricultural pest species *T. urticae* , where oxidative metabolism by P450s are clearly involved in Complex I inhibitor cross-resistance, although PBO synergism is not universal and other mechanisms, including target-site resistance are involved^15,17,37^.

The tolfenpyrad results were highly variable using the colonised strains. The control Kisumu strain had far lower mortality (15 ± 15%) than expected in the absence of PBO. This is most likely to be a function of the CDC bottle assays used and the chemical properties of tolfenpyrad (and to a lesser extent using fenpyroximate). At the high concentration of tolfenpyrad used, it seems likely that the compound crystalized under evaporation leading to uneven distribution in the bottle, resulting in lower potency and variability in results. In the presence of PBO, toxicity of tolfenpyrad was restored to the Kisumu strain, which may indicate that mixing of the Complex I inhibitor and PBO alters its surface distribution; PBO may thus act as an adjuvant as well as a synergist in this bioassay.

Lower than expected toxicity (87% ± 10) is also observed in the tolfenpyrad bioassays on control transgenic mosquitoes. Although these assays gave consistent results, it should be noted they were performed as tarsal bioassays, where the compounds are dried onto small glass plates and then mosquitoes exposed to the surface with limited space, so forcing interaction with the impregnated surface. Further consideration will be needed to determine the best method to screen future compounds for in vivo efficacy against pyrethroid resistant strains.

## Conclusions

Overall the study highlights the functionality of three compatible screening methods for the liabilities of new compounds to existing resistance mechanisms in mosquitoes. In vitro profiling of fenazaquin, pyridaben, tolfenpyrad and fenpyroximate flagged potential metabolic cross-resistance issues against *Anopheles* P450s commonly overexpressed in pyrethroid resistant populations of African mosquitoes. This was confirmed for tolfenpyrad and fenpyroximate, where in vivo killing efficacy was significantly reduced against pyrethroid resistant transgenic and laboratory strains of *Anophele*s. Together, the in vitro and in vivo data suggest that the Complex I inhibitors examined may not be effective for control of pyrethroid resistant mosquitoes due to their metabolic liability. Current trials of LLIN treated with PBO^38^ are investigating their potential to enhance the efficacy of conventional insecticides against pyrethroid resistant populations of *Anopheles* mosquitoes. It is likely that fenpyroximate and tolfenpyrad may also be more effective when used in combination with a P450 inhibitor such as PBO, which merits further investigation.

## Methods

### Reagents

Diethoxyfluoroscein (DEF) was purchased from Cypex Ltd, UK (www.cypex.co.uk). β-Nicotinamide adenine dinucleotide phosphate (NADP+) was purchased from Melford Laboratories Ltd, HPLC solvents from Fisher Scientific UK. Insecticides and all other reagents were supplied by Sigma Aldrich unless indicated otherwise.

### Insecticide metabolism assays

P450 activity is dependent on electrons donated by NADPH via CPR, thus metabolism was assessed by measuring insecticide turnover (percentage substrate depletion) in the presence or absence of NADPH. A cut-off value of 20% substrate depletion was used to distinguish turnover from baseline variability^24^. Reactions were also carried out in the presence and absence of cytochrome b5 (b5), which is located with P450 and CPR in the endoplasmic reticulum and can modify catalysis depending on the substrate and P450 involved^39^. Deltamethrin and DDT were included as comparative positive and negative controls, respectively, for P450 metabolism. Since this was designed as a rapid screen, the enzyme reactions were carried out using a single long time point of 2 h to detect a broad range of metabolic efficiency.

*E. coli* membranes co-expressing P450 and *An. gambiae* NADPH cytochrome P450 oxidoreductase (AgCPR) were supplied by Cypex Ltd, UK (www.cypex.co.uk). P450s were expressed using pCWori + expression vector constructs as described previously for CYPs 6M2 and 6P3, 6P4, 6P5, 9J5^20^ , CYP6Z2^26^, CYP6P9a^21^ and CYP9K1^25^. *An. gambiae* cytochrome b5 (b5) supplied by Cypex Ltd, UK was prepared as described previously to supplement enzyme reactions at a 8:1 molar ratio, b5:P450^40^. Reactions were carried out in vitro using a NADPH-regenerating system in triplicate in the presence and absence of NADP + . Insecticides were prepared as a working stock in ethanol and stored at − 20 °C; solvent content was 2% of the final reaction. A reaction volume of 100 µl contained 0.05 µM CYP450, 0.4 µM b5 (absent in − b5 reactions), 10 μM compound and 50 mM potassium phosphate buffer (KPB) at pH 7.4. The regenerating mix included 1 mM glucose-6-phosphate, 0.25 mM MgCl_2_, 0.1 mM NAPD + (absent –NADPH) and 1 unit/ml glucose-6-phosphate dehydrogenase (G6PDH). Reactions were pre-incubated at 30 °C for 5 min and started by adding the NADPH-regenerating mix, the reactions continued at 30 °C for 2 h, shaking at 1200 rpm and quenched with 100 µl acetonitrile. Samples were centrifuged at 16,000 *g* for 20 min, before 150 µl of the supernatant was used for HPLC. Results were calculated as percentage depletion of the insecticide peak area in the presence of NADPH (+NADPH) versus absence of NADPH (−NADPH) to give a quantitative assessment of metabolism.

### High-performance liquid chromatography (HPLC) analysis

Samples were analysed using a C18 Reverse-Phase LC Hypersil Gold Column, Thermofisher Scientific, on an Agilent 1100 series HPLC or Dionex UltiMate 3000 at 23 °C. 100 µl of organic solvent-quenched reaction supernatant was injected onto the column with isocratic mobile phases of 80% acetonitrile and 20% water with 0.1% phosphoric acid for fenpyroximate, pyridaben, tolfenpyrad, fenazaquin and deltamethrin, and 85% methanol and 15% water for DDT. Flow rates were 1 ml/min and monitoring absorbance wavelength was 226 nm apart from DDT, which was 232 nm. Elution times for the insecticides were as follows: fenpyroximate, 7.7 min; tolfenpyrad, 6.0 min; fenazaquin, 7.9 min; pyridaben, 9.3 min; deltamethrin, 9.8 min and DDT, 10.8 min. The insecticide was quantified by peak integration (OpenLAB Chromatography Data System). Examples of chromatograms used for peak quantification and quantitative assessment of P450 metabolism are illustrated in Supplementary Fig. 2.

### Rearing mosquitoes for bioassays

All bioassays were carried out on 2–5 day old non-blood-fed female mosquitoes, reared at the Liverpool School of Tropical Medicine (LSTM), in insectaries maintained at 26 °C ± 2 °C and 80% relative humidity ± 10% with a L12:D12 hour light:dark cycle and 1 h dawn and dusk^28^. Ground Tetramin tropical fish food flakes (Tetra, Blacksburg, VA, USA) was used as a larval diet, adults were provided with a 10% sucrose solution. For egg production, adult females adults were fed on 50:50 mixture of research red cells and plasma supplied by NHS Blood and Transplant, UK.

For the bioassays of P450 overexpressing mosquitoes, crosses were established between the homozygous ubiquitous Gal4 driver line Ubi-A10^22^, fluorescently tagged with CFP, and the homozygous responder lines UAS-Cyp6p3 and Cyp6m2, marked with yellow fluorescent protein^23^ or with wild type G3 mosquitoes (non-fluorescent). Pupal progeny were screened to confirm inheritance of correct markers and allowed to hatch into adults.

### Tarsal bioassays on transgenic mosquitoes

A glass plate tarsal contact assay described previously^8^ was used to expose transgenic adult females, alongside driver line/+ controls to 125 mg/m^2^ of each insecticide and 100 mg/m^2^ of the adjuvant RME (Mero, Bayer, Reading, UK: 81.4% w/w rapeseed fatty acid esters and emulsifier ethoxy (7) tridecanol) applied in acetone to glass Petri dishes (radius 2.5 cm, SLS, Nottingham, UK) for 30 min. Mosquitoes were then aspirated into holding cups and scored immediately for knock down, provided with 10% sugar solution on cotton wool and held at 27 ± 2 °C 70 ± 10% relative humidity 12:12 h light:dark cycle to be scored for mortality 24 and 48 h (± 2 h) after the end of exposure. Ten mosquitoes were exposed per glass plate, and 6 replicates were completed per treatment per strain.

### PBO assay

Three strains of *Anopheles gambiae*, Kisumu, Tiassalé 13 and VK7 2014, and one strain of *Anopheles funestus,* FUMOZ-R, were used for the in vivo PBO assays. Colonies were established, characterised and maintained as described by Williams et al.^28^.

The inside of 250 ml Wheaton bottles were coated according to the CDC ‘Guideline for Evaluating Insecticide Resistance in Vectors Using the CDC Bottle Bioassay’^29^ with 1.6 ml of solutions comprising insecticides dissolved in acetone plus the adjuvant RME, both with and without PBO, with 2 negative controls (acetone and RME, and acetone, RME and PBO) and 2 positive controls (20 µg/bottle permethrin, with and without PBO). The concentration of each test insecticide was the LC_95_ calculated from the results of dose response experiments with the Kisumu susceptible strain^8^: 160.8 µg/bottle Fenpyroximate and 143.84 µg/bottle Tolfenpyrad. A concentration of 400 µg/bottle PBO was used as it is recommended by the CDC for synergist bioassays^41^. Three replicate bottles were coated and tested per treatment for each of the four strains.

Bioassays were conducted in controlled conditions: 27 ± 2 °C 70 ± 10% relative humidity. Twenty five 2–5 day old adult female mosquitoes, allowed to mate but not blood feed, were added to each bottle and left for a 60 min exposure period. Mosquitoes were then aspirated into holding cups and scored immediately for knock down, provided with 10% sugar solution on cotton wool and held at 27 ± 2 °C 70 ± 10% relative humidity 12:12 h light:dark cycle to be scored for mortality 24 and 48 h (± 2 h) after the end of exposure. Three replicate bioassays were carried out per strain, each with a different set of bottles coated no more than 24 h previously. Knock down was scored immediately after the one hour exposure and mortality was scored 24 and 48 h post-exposure as defined by the World Health Organisation (‘immobile or unable to stand or take off’)^28^.

### Data analysis

In the transgenic assays mean mortality 24 h post-exposure was calculated from all replicates of a treatment, reported ± 95% confidence intervals. Where 95% CI for a strain overexpressing a P450 did not overlap with the mean mortality in the control group the effect of P450 overexpression on insecticide sensitivity was deemed to be significant.

In the PBO assay, to detect cross resistance in the pyrethroid resistant strains, mean mortality 24 h post-exposure was calculated from all replicates of a treatment, reported ± 95% confidence intervals. Where 95% CI for a resistant strain did not overlap with the mean mortality for Kisumu the level of cross resistance was deemed to be significant.

The effect of PBO on 24-h mortality for each compound was assessed, and pairwise comparisons made between observations at different time points, using a 1 tailed Fisher’s Exact test to determine where synergism was significant (p < 0.001).

### Supplementary information

Supplementary Information.

## Data Availability

Raw data files are available upon request.

## References

[1] Bhatt S (2015). The effect of malaria control on *Plasmodium falciparum* in Africa between 2000 and 2015. Nature.

[2] Hemingway J (2016). Averting a malaria disaster: Will insecticide resistance derail malaria control?. Lancet.

[3] Toé KH (2014). Increased pyrethroid resistance in malaria vectors and decreased bed net effectiveness, Burkina Faso. Emerg. Infect. Dis..

[4] World Health Organization. Global report on insecticide resistance in malaria vectors: 2010–2016. World Health Organization. https://apps.who.int/iris/handle/10665/272533 (2018)

[5] Silva APB, Santos JMM, Martins AJ (2014). Mutations in the voltage-gated sodium channel gene of anophelines and their association with resistance to pyrethroids—A review. Parasit. Vect..

[6] David JP, Ismail HM, Chandor-Proust A, Paine MJI (2013). Role of cytochrome P450s in insecticide resistance: Impact on the control of mosquito-borne diseases and use of insecticides on Earth. Philos. Trans. R. Soc. Lond. B. Biol. Sci..

[7] Hoppé M, Hueter OF, Bywater A, Wege P, Maienfisch P (2016). Evaluation of commercial agrochemicals as new tools for malaria vector control. Chim. Int. J. Chem..

[8] Lees R (2019). A testing cascade to identify repurposed insecticides for next-generation vector control tools: Screening a panel of chemistries with novel modes of action against a malaria vector. Gates Open Res..

[9] Pridgeon JW (2008). Susceptibility of Aedes aegypti, culex quinquefasciatus Say, and Anopheles quadrimaculatus say to 19 pesticides with different modes of action. J. Med. Entomol..

[10] Hueter OF, Hoppé M, Wege P, Maienfisch P (2016). Identification and optimization of new leads for malaria vector control. Chimia (Aarau)..

[11] Hollingworth RM, Ahammadsahib KI, Gadelhak G, McLaughlin JL (1994). New inhibitors of Complex I of the mitochondrial electron transport chain with activity as pesticides. Biochem. Soc. Trans..

[12] Lümmen P (1998). Complex I inhibitors as insecticides and acaricides. Biochim. Biophys. Acta - Bioenerg..

[13] Stewart ZP (2013). Indoor application of attractive toxic sugar bait (ATSB) in combination with mosquito nets for control of pyrethroid-resistant mosquitoes. PLoS ONE.

[14] Malima R (2017). Experimental hut evaluation of a novel long-lasting non-pyrethroid durable wall lining for control of pyrethroid-resistant Anopheles gambiae and Anopheles funestus in Tanzania. Malar. J..

[15] Van Leeuwen T, Vontas J, Tsagkarakou A, Dermauw W, Tirry L (2010). Acaricide resistance mechanisms in the two-spotted spider mite Tetranychus urticae and other important Acari: A review. Insect Biochem. Mol. Biol..

[16] Stumpf N, Nauen R (2001). Cross-resistance, inheritance, and biochemistry of mitochondrial electron transport inhibitor-acaricide resistance in Tetranychus urticae (Acari: Tetranychidae). J. Econ. Entomol..

[17] Van Pottelberge S, Van Leeuwen T, Nauen R, Tirry L (2009). Resistance mechanisms to mitochondrial electron transport inhibitors in a field-collected strain of Tetranychus urticae Koch (Acari: Tetranychidae). Bull. Entomol. Res..

[18] Riga M (2015). Functional characterization of the Tetranychus urticae CYP392A11, a cytochrome P450 that hydroxylates the METI acaricides cyenopyrafen and fenpyroximate. Insect Biochem. Mol. Biol..

[19] Ranson H, Lissenden N, Lissenden A (2016). Insecticide resistance in African Anopheles mosquitos: A worsening situation that needs urgent action to maintain malaria control. Trends Parasitol..

[20] Yunta C (2016). Pyriproxyfen is metabolized by P450s associated with pyrethroid resistance in An. gambiae. Insect Biochem. Mol. Biol..

[21] Yunta C (2019). Cross-resistance profiles of malaria mosquito P450s associated with pyrethroid resistance against WHO insecticides. Pestic. Biochem. Physiol..

[22] Adolfi A, Pondeville E, Lynd A, Bourgouin C, Lycett GJ (2018). Multi-tissue GAL4-mediated gene expression in all Anopheles gambiae life stages using an endogenous polyubiquitin promoter. Insect Biochem. Mol. Biol..

[23] Adolfi A (2019). Functional genetic validation of key genes conferring insecticide resistance in the major African malaria vector Anopheles gambiae. Proc. Natl. Acad. Sci..

[24] Jones HM, Houston JB (2004). Substrate depletion approach for determining in vitro metabolic clearance: Time dependencies in hepatocyte and microsomal incubations. Drug Metab. Dispos..

[25] Vontas J (2018). Rapid selection of a pyrethroid metabolic enzyme CYP9K1 by operational malaria control activities. Proc. Natl. Acad. Sci..

[26] Mclaughlin LA (2008). Characterization of inhibitors and substrates of Anopheles gambiae CYP6Z2. Insect Mol Biol.

[27] Chandor-Proust A (2013). The central role of mosquito cytochrome P450 CYP6Zs in insecticide detoxification revealed by functional expression and structural modelling. Biochem. J..

[28] Williams J (2019). Characterisation of Anopheles strains used for laboratory screening of new vector control products Parasites and Vectors. Parasit. Vec..

[29] Brogdon W, Chan A (2010). Guideline for Evaluating Insecticide Resistance in Vectors Using the CDC Bottle Bioassay.

[30] World Health Organization. *Test procedures for insecticide resistance monitoring in malaria vector mosquitoes*. (2016).

[31] Schenkman JB, Jansson I (2003). The many roles of cytochrome b5. Pharmacol. Ther..

[32] Finn RD (2008). Defining the in vivo role for cytochrome b5 in cytochrome P450 function through the conditional hepatic deletion of microsomal cytochrome b5. J. Biol. Chem..

[33] Ismail HM (2013). Pyrethroid activity-based probes for profiling cytochrome P450 activities associated with insecticide interactions. Proc. Natl. Acad. Sci. USA..

[34] Ingham VA (2020). A sensory appendage protein protects malaria vectors from pyrethroids. Nature.

[35] Riveron JM (2013). Directionally selected cytochrome P450 alleles are driving the spread of pyrethroid resistance in the major malaria vector Anopheles funestus. Proc. Natl. Acad. Sci. USA.

[36] Menze BD (2016). Multiple insecticide resistance in the malaria vector Anopheles funestus from Northern Cameroon is mediated by metabolic resistance alongside potential target site insensitivity mutations. PLoS ONE.

[37] Bajda S (2017). A mutation in the PSST homologue of complex I (NADH:ubiquinone oxidoreductase) from Tetranychus urticae is associated with resistance to METI acaricides. Insect Biochem. Mol. Biol..

[38] Staedke SG (2019). LLIN evaluation in Uganda Project (LLINEUP)—Impact of long-lasting insecticidal nets with, and without, piperonyl butoxide on malaria indicators in Uganda: Study protocol for a cluster-randomised trial. Trials.

[39] Barnaba C, Gentry K, Sumangala N, Ramamoorthy A (2017). The catalytic function of cytochrome P450 is entwined with its membrane-bound nature. F1000Research.

[40] Stevenson BJ (2011). Cytochrome P450 6M2 from the malaria vector Anopheles gambiae metabolizes pyrethroids: Sequential metabolism of deltamethrin revealed. Insect Biochem. Mol. Biol..

[41] Centers for Disease Control and Prevention Guideline for evaluating insecticide resistance in vectors using the CDC bottle bioassay. https://www.cdc.gov/malaria/resources/pdf/fsp/ir_manual/ir_cdc_bioassay_en.pdf

