# Author Correction: New insecticide screening platforms indicate that Mitochondrial Complex I inhibitors are susceptible to cross-resistance by mosquito P450s that metabolise pyrethroids

**DOI:** 10.1038/s41598-023-43915-z

**Published:** 2023-10-06

**Authors:** Rosemary S. Lees, Hanafy M. Ismail, Rhiannon A. E. Logan, David Malone, Rachel Davies, Amalia Anthousi, Adriana Adolfi, Gareth J. Lycett, Mark J. I. Paine

**Affiliations:** 1 Vector Biology Department, Liverpool School of Tropical Medicine, Liverpool, L3 5QA UK; 2 Innovative Vector Control Consortium, Liverpool School of Tropical Medicine, Liverpool, L3 5QA UK

Correction to: *Scientific Reports* 10.1038/s41598-020-73267-x, published online 01 October 2020

The Acknowledgements section in the original version of this Article was incomplete.

“The work was funded by the IVCC.”

now reads:

“The work was funded by the IVCC (supported by the Bill and Melinda Gates Foundation under Grant Number OPP1148615), and Medical Research Council (Grant Ref: MR/V001264/1).”

The original Article has been corrected.

