# Supplementary material for: New insecticide screening platforms indicate that Mitochondrial Complex I inhibitors are susceptible to cross-resistance by mosquito P450s that metabolise pyrethroids
Source: Sci Rep. 2020 Oct 1;10:16232. doi: 10.1038/s41598-020-73267-x (PMC7530702; doi:10.1038/s41598-020-73267-x)

**Supplementary Information**

**Title: New insecticide screening platforms indicate that Mitochondrial Complex I inhibitors are susceptible to cross-resistance by mosquito P450s that metabolise pyrethroids**

Rosemary S. Lees^1,3^, Hanafy M. Ismail^1,3^, Rhiannon A. E. Logan^1^, David Malone^2^, Rachel Davies^1^, Amalia Anthousi^1^, Adriana Adolfi^1^, Gareth J. Lycett^1*^, & Mark J.I. Paine^1^*

**Affiliations:**

^1^ Vector Biology Department, Liverpool School of Tropical Medicine, Liverpool, L3 5QA, UK

^2^ IVCC, Liverpool School of Tropical Medicine, Liverpool, L3 5QA, UK

^3^ These authors contributed equally

* Coresponding authors, [mark.paine@lstmed.ac.uk](mailto:mark.paine@lstmed.ac.uk) and gareth.lycett@lstmed.ac

**Supplementary Table 1**. Insecticide metabolism by mosquito P450s

| **P450** | **+/- b5** | **% Insecticide Depletion*** | | | | | |
| --- | --- | --- | --- | --- | --- | --- | --- |
|  |  | **Deltamethrin** | **DDT** | **Tolfenpyrad** | **Fenazaquin** | **Fenpyroximate** | **Pyridaben** |
| 6P3 | + | 91.4 ± 6.5^b^ | 3.8 ± 8.3 | 97.7 ± 0.5^b^ | 97.5 ± 0.7 ^b^ | 65.36 ± 3.45 ^b^ | 98.28 ± 0.20^c^ |
|  | - | 76.9 ± 4.6^c^ | 0.00 | 98.5 ± 0.17 ^b^ | 92.64 ± 2.7 ^c^ | 12.78 ± 0.73 ^a^ | 77.59 ± 0.87^b^ |
| 6P9a | + | 48.62 ± 1.85^a^ | 0.00 | 70.3 ± 0.79 ^c^ | 93.4 ± 2.89 ^c^ | 88.3 ± 1.66 ^c^ | 89.05 ± 6.97^b^ |
|  | - | 48.51 ± 2.16^c^ | 0.00 | 60.6 ± 0.63 ^c^ | 96.8 ± 0.47 ^c^ | 11.26 ± 6.94 | 61.97 ± 1.68^b^ |
| 6M2 | + | 32.1 ± 0.63^b^ | 3.4 ± 1.17^a^ | 57.16 ± 0.87 ^b^ | 67.17 ± 0.6 ^c^ | 77.3 ± 0.3 ^c^ | 60.15 ± 2.11^b^ |
|  | - | 25.6 ± 2.53^c^ | 2.6 ± 4.58 | 43.44 ± 1.9 ^c^ | 17.71 ± 5.7 ^c^ | 4.91 ± 1.57 | 34.82 ± 3.75^b^ |
| 6Z2 | + | 8.53 ± 0.99^b^ | 0.56 ± 7.47 | 12.1 ± 2.54 ^c^ | 72.4 ± 0.24 ^c^ | 27.6 ± 2.66 ^c^ | 13.32 ± 6.43^a^ |
|  | - | 6.05 ± 0.82^b^ | 0.12 ± 7.08 | 21.6 ± 2.85 ^c^ | 50.6 ± 3.15 ^b^ | 29.1 ± 0.83 ^b^ | 26.49 ± 22.94^ns^ |
| 6P4 | + | 41.96 ± 1.70^c^ | 2.16 ± 1.38 | 98.4 ± 0.59 ^c^ | 92.35 ± 1.47 ^a^ | 90.8 ± 2.25 ^c^ | 94.32 ± 1.74 ^b^ |
|  | - | 36.43 ± 2.50^b^ | 0.00 | 96.60 ± 2.38 ^b^ | 48.48 ± 1.55 ^b^ | 17.86 ± 4.98 ^a^ | 78.26 ± 0.24 ^b^ |
| 9J5 | + | 25.61 ± 2.09^c^ | 0.73 ± 5.67 | 32.6 ± 0.88 ^c^ | 33.70 ± 3.25 ^b^ | 29.9 ± 3.27 ^c^ | 59.70 ± 0.13 ^c^ |
|  | - | 16.54 ± 1.92^b^ | 0.00 | 24.23 ± 6.86 ^b^ | 13.41 ± 5.31 | 15.78 ± 5.50 ^a^ | 38.09 ± 2.79 ^b^ |
| 6P2 | + | 91.94 ± 0.52^c^ | 5.2 ± 1.6 | 91.9 ± 0.52 ^c^ | 88.8 ** | 23.67 ± 1.53 ^b^ | 74.50 ± 22.08 ^a^ |
|  | - | 94.20 ± 0.55^c^ | nd | 94.2 ± 0.55 ^c^ | 49.81** | 7.56 ± 1.45 ^b^ | 25.76 ± 8.40 ^b^ |
| 9K1 | + | 14.29 ± 5.77^b^ | 0.00 | 53.19 ± 4.27 ^b^ | 22.8 ± 2.82 ^c^ | 12.80 ± 3.05 ^a^ | 60.45 ± 3.97 ^b^ |
|  | - | 17.80 ± 5.10^b^ | 1.83 ± 3.74 | 22.38 ± 0.77 ^b^ | 17.0 ± 1.14 ^ns^ | 11.18 ± 3.75 ^ns^ | 49.12 ± 9.50 ^a^ |

* mean ± SD; nd, not determined; significantly greater insecticide clearance compared to negative reactions (no NADPH supplied) are indicated  ^a^ P<0.05, ^b^ P<0.01, ^c^  P<0.001 (paired T-test); **mean of two experiments

**Supplementary Table 2** Average knock down at the end of exposure and mortality 24- and 48-hour post-exposure of adult transgenic female *Anopheles* mosquitoes to fenpyroximate or tolfenpyrad or in a glass plate tarsal assay.

| Insecticide (125 mg/m^2^) | Strain | Average KD (%) | Average 24h mortality (%) | Average 48h mortality (%) |
| --- | --- | --- | --- | --- |
| Fenpyroximate | Ubi-A10/+ | 100 | 100 | 100 |
|  | Ubi-A10/P3 | 100 | 95 | 93 |
|  | Ubi-A10/M2 | 59 | 28 | 40 |
| Tolfenpyrad | Ubi-A10/+ | 98 | 88 | 86 |
|  | Ubi-A10/P3 | 95 | 32 | 36 |
|  | Ubi-A10/M2 | 74 | 35 | 37 |

**Supplementary Table 3.** Average knock down at the end of exposure and mortality 24 and 48 hours post-exposure of adult female *Anopheles* mosquitoes to fenpyroximate, tolfenpyrad or permethrin alone or with the addition of PBO in a CDC bottle bioassay.

| Insecticide | PBO | Colony | Average KD (%) | Average 24h mortality (%) | Average 48h mortality (%) |
| --- | --- | --- | --- | --- | --- |
| Fenpyroximate (160.8 µg per bottle) | - | Kisumu | 73.7 | 73.7 | 79.8 |
|  |  | Tiassalé 13 | 51.3 | 42.3 | 33.1 |
|  |  | VK7 2014 | 23.4 | 12.6 | 14.1 |
|  |  | FUMOZ-R | 19.7 | 5.8 | 8.6 |
|  | + | Kisumu | 100.0 | 100.0 | 100.0 |
|  |  | Tiassalé 13 | 86.4 | 71.3 | 80.5 |
|  |  | VK7 2014 | 88.7 | 69.6 | 72.2 |
|  |  | FUMOZ-R | 86.7 | 98.5 | 100.0 |
| Tolfenpyrad (143.84 µg per bottle) | - | Kisumu | 50.5 | 15.5 | 22.9 |
|  |  | Tiassalé 13 | 61.4 | 61.4 | 52.1 |
|  |  | VK7 2014 | 8.9 | 8.1 | 6.2 |
|  |  | FUMOZ-R | 11.1 | 2.8 | 5.6 |
|  | + | Kisumu | 92.3 | 92.1 | 97.3 |
|  |  | Tiassalé 13 | 79.7 | 94.6 | 94.2 |
|  |  | VK7 2014 | 87.0 | 95.7 | 97.1 |
|  |  | FUMOZ-R | 93.3 | 79.5 | 84.8 |
| Permethrin  (20 µg per bottle) | - | Kisumu | 99.3 | 97.9 | 100.0 |
|  |  | Tiassalé 13 | 41.3 | 64.2 | 62.8 |
|  |  | VK7 2014 | 15.6 | 8.2 | 16.9 |
|  |  | FUMOZ-R | 97.2 | 59.4 | 60.0 |
|  | + | Kisumu | 100.0 | 100.0 | 100.0 |
|  |  | Tiassalé 13 | 97.1 | 100.0 | 98.8 |
|  |  | VK7 2014 | 63.8 | 70.6 | 74.4 |
|  |  | FUMOZ-R | 100.0 | 94.9 | 97.1 |

**Supplementary Figure 1.** **Mortality in 4 strains of *Anopheles* females 24 hours after exposure in a CDC bottle bioassay to permethrin or acetone-only negative control treatments, alone or with the addition of PBO.** Female adults were exposed to the inside of a glass bottle, around 25 adults per bottle and 3 replicate bottle per treatment and strain. Bottles were coated with acetone alone or 20 µg per bottle of permethrin in acetone, with or without 400 µg of PBO. Box plots represent the median (centre line), 25^th^ and 75^th^ percentiles (box) and upper and lower adjacent values (whiskers) of 3 replicates, and dots represent outlier values.

**Supplementary Figure 2. Representative HPLC chromatograms of Complex I and control compounds.** The overlaid chromatograms represent the results of 2 hour incubations of 100 μl reactions containing 0.05 μM CYP6M2 0.4 μM b5 and 10 μM compound in the presence (black) and absence (blue) of NADPH. Substrate peaks are arrowed.


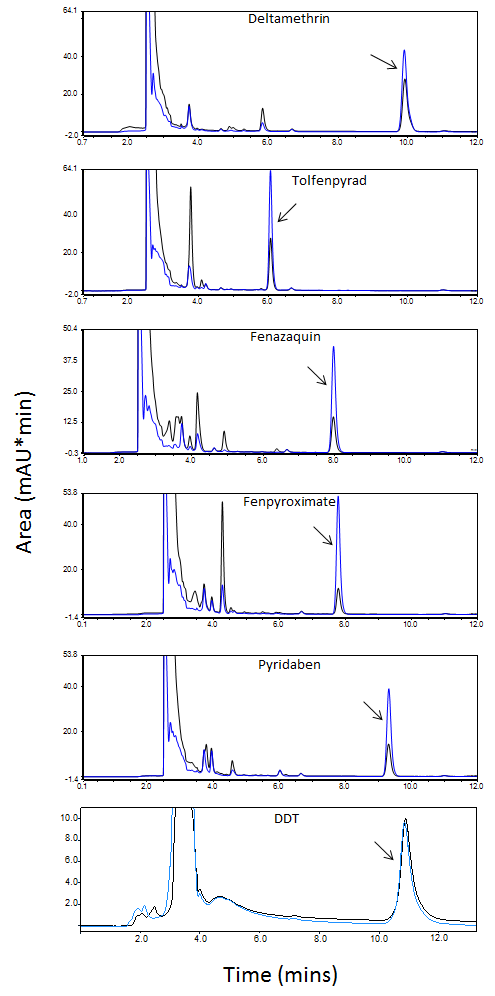

Supplement: Supplementary file 1 — Supplementary Information. [file 41598_2020_73267_MOESM1_ESM.docx]
